# Exploration of Tools for the Interpretation of Human Non-Coding Variants

**DOI:** 10.3390/ijms232112977

**Published:** 2022-10-26

**Authors:** Nicole Tabarini, Elena Biagi, Paolo Uva, Emanuela Iovino, Tommaso Pippucci, Marco Seri, Andrea Cavalli, Isabella Ceccherini, Marta Rusmini, Federica Viti

**Affiliations:** 1 Computational and Chemical Biology, Italian Institute of Technology, 16163 Genoa, Italy; 2 Department of Medical and Surgical Sciences, University of Bologna, 40138 Bologna, Italy; 3 Clinical Bioinformatics, IRCCS Istituto Giannina Gaslini, 16147 Genoa, Italy; 4 Medical Genetics Unit, IRCCS Sant’Orsola-Malpighi University Hospital, 40138 Bologna, Italy; 5 Department of Pharmacy and Biotechnology, University of Bologna, 40126 Bologna, Italy; 6 UOSD Laboratory of Genetics and Genomics, IRCCS Istituto Giannina Gaslini, 16147 Genoa, Italy; 7 Istituto di Biofisica, Consiglio Nazionale delle Ricerche, 16149 Genoa, Italy

**Keywords:** whole genome sequencing, non-coding variants, variant prioritization, 5′UTR, splicing prediction, mutation intolerance

## Abstract

The advent of Whole Genome Sequencing (WGS) broadened the genetic variation detection range, revealing the presence of variants even in non-coding regions of the genome, which would have been missed using targeted approaches. One of the most challenging issues in WGS analysis regards the interpretation of annotated variants. This review focuses on tools suitable for the functional annotation of variants falling into non-coding regions. It couples the description of non-coding genomic areas with the results and performance of existing tools for a functional interpretation of the effect of variants in these regions. Tools were tested in a controlled genomic scenario, representing the ground-truth and allowing us to determine software performance.

## 1. Introduction

The sequencing of the whole human genome, completed in 2003, carried the belief of easily solving the genetic ground of many diseases. If this has been proved to be true for genetic disorders caused by mutations in coding sequences, it took many years and efforts to understand the importance and the large involvement in diseases of the non-coding regions, long referred to as ‘junk DNA’. Currently, it is known that around 80% of the human genome contains functional elements [1], that up to 5–10% is under purifying selection [1,2], that non-coding elements are crucial to controlling the expression of protein-coding genes [3], and that variants in non-coding portions (non-coding variants, NCVs) of the genome can have a critical impact on perturbing gene regulation, thus potentially causing a variety of diseases. Moreover, recent studies have revealed an implication of non-coding variants in regulating DNA replication timing [4], suggesting an indirect link between DNA synthesis and transcription [5]. In this complex scenario, deciphering the roles of non-coding variants in disease etiology remains nontrivial [3].

Whole Genome Sequencing (WGS) is potentially able to reveal single-nucleotide variants (SNVs), insertion/deletions (InDels), as well as structural variants (SVs) and copy-number variants (CNVs) throughout the whole genome [6], thus allowing the detection of causative disease variants which could be missed by targeted approaches. However, the potential of WGS is not fully exploited because the current knowledge of non-coding regions is still lagging behind coding ones. Ellingford et al. [7] report that 63.4% of untranslated region (UTR) variants in the ClinVar database are categorized as ‘variants of uncertain significance’ (VUS), thus demonstrating that non-coding regions are still widely under-ascertained in clinical databases, even when representing quite well-studied areas. In this context, novel algorithms are continuously being proposed to predict the pathogenicity of variants in such critical regions, and reviews are constantly published trying to shed light on the selection of best practices and methods to maximize successful results. For example, Wang et al. [8] compared 24 existing methods to predict the effects of variants in the human non-coding sequences, showing that current methods provide better concordance in results when analyzing somatic variants than when dealing with germinal ones. On the other hand, they demonstrate that existing computational methods show acceptable performance only for germline variants, and that their predictive ability should be improved for different types of non-coding variants. Even Rojano et al. [9] worked on a promising set of tools for predicting the potential impact of variants falling in regulatory regions of the genome, showing the advantages and limitations of tools dedicated to variant annotation more than to variant effect interpretation. Although imperfect and in-progress, prediction tools currently represent the unique recognized way and necessary step for rapid and massive interpretation of ‘non-coding variants’. Nevertheless, they could hide pitfalls and issues and often require functional tests, such as RNA sequencing or targeted approaches, multiplexed assays of variant effects, chromatin interaction assays, and reporter gene assays to validate predictions [7]. Several other reviews have been published describing the limits and strengths of different tools [10,11], focusing on those currently available to visualize and functionally annotate WGS variants without performing comparative evaluation and benchmarking. In this review, we provide an updated critical overview of the currently available bioinformatics tools for interpreting SNP/indel falling in non-coding regions (NCVs from now on), grouping selected methods based on the non-coding regions they can target. For each of them, we: quantitatively assess and compare the performance of the algorithms at computational and information levels, also evaluating computational resources required for the execution; provide knowledge on their usability and effectiveness in different genomic contexts; critically highlight limitations and advantages, when additional to those intrinsically residing in our inclusion criteria (described in the following). To group tools depending on their target region, recent suggestions for functional element identification were followed [12].

## 2. Methods

### 2.1. Inclusion Criteria

For each considered tool, specific features such as input requirements, output format, supported reference genome, and the genomic context suitable for the tool are reported. According to these criteria, methods were classified into three main categories: aggregators, integrators, and investigators. In particular, software solutions that collect information on variants’ annotation from other tools to propose a comprehensive overview of the knowledge related to variants in non-coding regions were defined as ‘aggregators’. Tools that combine variant properties (e.g., regulatory features, conservation metrics, genic context) to provide refined scores for classifying or prioritizing the potential impact of such variants were named ‘integrators’. Tools that develop ex-novo algorithms to investigate and explain the functional consequences of variants in non-coding regulatory elements were referred to as ‘investigators’. The ‘investigator’ tools, which not only annotate the predicted impact of the variants in non-coding regions but can also provide information concerning the functional effects of the variants, represent the focus of this review. Among them, we identified 10 methods suitable for being included in bioinformatics pipelines for WGS data analysis. Selection was performed based on the following criteria: (i) being freely available; (ii) accepting VCF files as input; (iii) being fully accessible, including all additional datasets necessary for running the tool. Selected programs were benchmarked based on their intended usage in a controlled scenario, enabling the assessment of quantitative performance. Programs were executed using hg19 as the reference genome.

### 2.2. The Controlled Scenario: Knowledge-Based and Computational Performance

A set of manually curated known pathogenic and benign NCVs was obtained from ncVarDB [13]. ncVarDB includes 721 certainly pathogenic and 7228 certainly benign NCVs, spread over the whole human genome. Since it relies on the hg38 reference genome, coordinates were converted to hg19 using the liftOver tool [14]. After conversion, three benign variants (rs412762, rs878890367, and rs3208965) were lost. The number of SNPs in each class (intronic, intergenic, ncRNA, 3′UTR, and 5′UTR) is reported in Table 1.

To evaluate the computational resources required by the tools, we in silico merged the set of known variants with the variants in chromosome 20 from a sample of Han Chinese ancestry [15] (HG005-NA24631) retrieved from the Genome In A Bottle (GIAB) project [16]. Chromosome 20 (64,444,167 bp) contained 78,186 variants distributed as follows: 88.34% single nucleotide variants (SNVs), 11.2% small insertions/deletions (InDels), and 0.46% larger sequence alterations; the set of non-coding variants (99.37%) overcame by far the coding and splicing ones (0.52% and 0.11%, respectively).

The merged dataset contained 86,132 mutations and allowed us to benchmark the performance in pathogenicity prediction and computational efficiency in a reasonable time-frame.

All variants were annotated with VEP to identify the genomic region where they are mapped [17]. For each tool, a set of performance metrics was retrieved including: (i) the number of variants they could annotate; (ii) the computational time, calculated by running jobs on a single node, with 2 12-cores CPUs (24 total cores); (iii) the availability of parallel paradigm in the software; (iv) specificity = TN/[TN + FP]; (v) precision = TP/[TP + FP]; when allowed, (vi) sensitivity = TP/[TP + FN]; (vii) accuracy = [TP + TN]/[TP + TN + FP + FN]. The last 4 metrics rely on the confusion matrices obtained comparing predicted results to the ncVarDB knowledge base. In detail, (i) true positives (TP) correspond to variants labeled as pathogenic by the tool under analysis AND by ncVarDB, (ii) true negatives (TN) refer to variants labeled as benign by the tool AND by ncVarDB, (iii) false positives (FP) represent variants labeled as pathogenic by the tool BUT as benign by ncVarDB, and (iv) false negatives (FN) are variants that, although pathogenic for ncVarDB, are not called pathogenic by the tool. In all the cases, except for the UTRs-related tools, it was not possible to calculate FN, as the ability of the studied tool to evaluate the biological function responsible for pathogenicity could not be clearly assessed. For example, if a deep intronic variant has not been classified as pathogenic by splicing tools, it should not be considered a false negative since its pathogenicity could still be attributed to a function other than splicing.

## 3. Results

### 3.1. Method Selection

**Figure 1:**
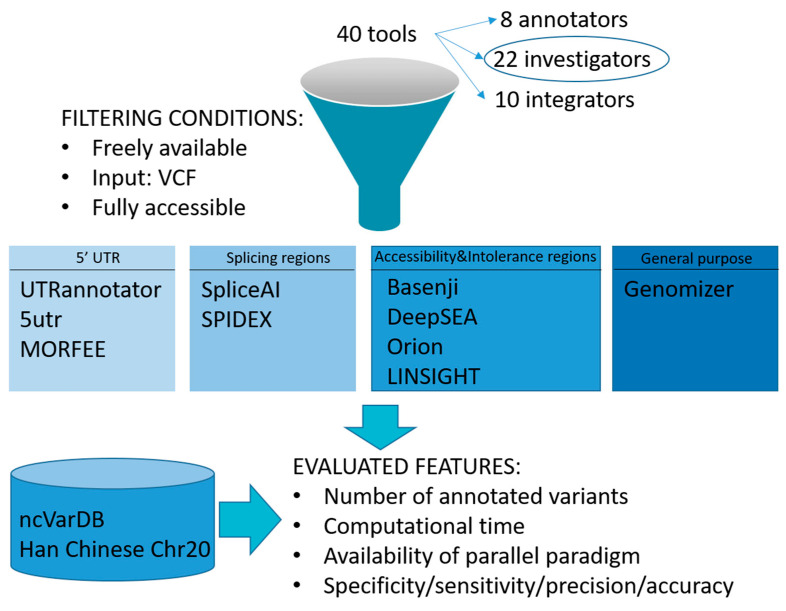
Graph of the approach followed in defining, grouping, and analyzing selected tools. A first literature screening was performed to retrieve the most promising existing methods for NCVs analysis. They belong to ‘Annotators’, ‘Integrators’, or ‘Investigators’ groups. Focusing on components of the ‘Investigators’ group, inclusion criteria were applied to select a set of best-hits. They were clustered relying on the genomic region they targeted, and several features were critically evaluated.

A total of 40 tools for NCV interpretation were initially considered (Table 2). Of these, 10 ‘investigator’ tools passed our inclusion criteria (see ‘Inclusion criteria’ section for details) and were retained for in-depth evaluation: Basenji, DeepSEA, Genomiser, LINSIGHT, MORFEE, Orion, SPIDEX, SpliceAI, 5utr, UTRannotator (Figure 1). Their performance (Table 3) was evaluated and described in the genomic context specific to each tool, except for Genomiser, which was considered separately.

### 3.2. Tools for Identifying NCV Affecting UTRs

Messenger RNA (mRNA) is surrounded by transcribed but untranslated regions (UTRs), namely 5′ (upstream) and 3′ (downstream) UTRs. These intervene in the post-transcriptional phase contributing to the regulation of mRNA stability, secondary structure, localization, and translation. Notably, 3.7% of the genetic variants detected in GWAS studies are located in UTRs [56,57]. Moreover, 3′UTRs usually carry polymorphisms (MAF > 1%), whereas mutations (MAF < 1%) predominate in 5′UTR [58]. The 3′UTR is characterized by binding sites for RNA-binding proteins (RBPs) and microRNAs (miRNA); thus, variants in this region may lead to unstable mRNAs by creating or abolishing critical binding sites for long non-coding RNAs, miRNAs, or proteins involved in the regulation of translation. 5′UTR should possess a Kozak consensus sequence (ACCAUGG), which contains the translation initiation codon and other regulatory elements like CpG sites, uORFs (upstream Open Reading Frame), IRESs (internal ribosome entry sites), and RBP binding sites. Moreover, it could house secondary structures, such as hairpins, which are important in translational regulation [59]. Variations in length and sequence in 5′UTR could have a significant impact on the overall production of the protein, mainly causing: (i) the establishment of upstream start sites (uAUGs); (ii) the disruption of the pre-existing start or stop codons of uORFs which are crucial tissue-specific cis regulators of translation and are present in around half of all gene 5′UTRs; (iii) a frameshift in an existing uORF. A recent study demonstrated that variants in 5′UTRs creating new upstream start codons or disrupting stop codons of existing uORFs are under strong negative selection [60].

Currently, no tool is available specifically for NCV in 3′UTRs, while three ‘investigator’ tools for 5′UTR variants were identified as representative of the pool of methods targeting this region: *UTRannotator*, *5utr*, *MORFEE*.

***UTRannotator* [36]** is a comprehensive tool for studying variants impacting uORFs in eukaryotes. For each 5′UTR variant provided in the VCF input file, *UTRannotator* evaluates whether it could lead to any of the effects listed in the previous paragraph, assigning the number of overlapping ORFs within the 5′UTR, both in-frame and out-of-frame, and counting 5′UTR-uORFs with a stop codon in the reference sequence. For each of these variants, transcript-specific annotation is provided, leading to multiple annotation consequences. As output, *UTRannotator* supports default VEP output, tab-delimited, and VCF formats. For each of the five different conditions (uAUG gained/lost, uSTOP gained/lost, uFrameShift), the tool evaluates the type of uORF disrupted and/or created (e.g., inframe, OutOfFrame), the Kozak consensus sequence [61] and strength, as well as other features [62]. *UTRannotator* is available as a VEP plugin, which represents an advantage to VEP users.

In the reference dataset, *UTRannotator* could recognize 718 variants, resulting in 727 annotations (some variants were located in the 5′UTR of multiple genes, e.g., rs12974606). Considering the 74 variants predicted to have a pathogenic effect on 5′UTR, ‘uAUG gained’ was the most represented class of uORF-perturbing consequence (26 variants), followed by ‘uAUG lost’ (18 variants), ‘uFrameShift’ (11 variants), ‘uSTOP gained’ (7 variants), and ‘uSTOP lost’ (7 variants). Peculiar effects were highlighted for 5 specific variants, such as ‘uAUG gained & uAUG lost’. The main limitations of *UTRannotator* are the constraint to work on variants shorter than 5 bps, and the consideration of only canonical AUG start sites. 

***5utr* [63]**, also available as a VEP plugin, making it easy to run for VEP users, retrieves the distance of the variant from the main start codon of the coding sequence (CDS), the information about AUG and STOP loss/gain/change and auxiliary fields (e.g., in/out of frame and Kozak sequence and strength) noteworthy for the interpretation of variant effects. In particular, TE_log2fold [64] indicates the change in predicted translational efficiency caused by the variant. Delta_dsRNA and Delta_G4 [65] parameters indicate the change in minimum free energy of the double-stranded RNA (secondary structure) and quadruplex structures, respectively, representing their stability. The increased stability of secondary structures due to the variants is indicated by Delta_dsRNA negative values, which are associated with reduced translation. The VEP-based annotation resulted in 718 variants falling in 5′UTRs (727 annotations, considering variants located in the 5′UTR of multiple genes), where 151 were identified with a pathogenic effect. Among these, *5utr* recognised 14 ‘STOP_change’, 33 ‘STOP_loss’, 57 ‘STOP_gain’, 1 ‘AUG_change’, 21 ‘AUG_gain’, 12 ‘AUG_loss’, 3 ‘AUG_loss & STOP_change’, 2 ‘AUG_gain & STOP_change’, 2 ‘AUG_loss & STOP_loss’, 4 ‘AUG_gain & STOP_gain’, and 2 ‘AUG_loss & STOP_gain’ events. 

A ‘delta_G4’ score (ΔG4) = 0 was assigned to 260 variants (94.2%), 6 variants had a ΔG4 < 0, two had 0 < ΔG4 < 1 and 2 variants obtained a ΔG4 = 1. Of the remaining 6 variants presenting a ΔG4 > 1, the highest ΔG4 value was 12.5. Concerning ‘delta_dsRNA’ (ΔRNA), 41 variants were assigned a ΔRNA = 0; 126 were attributed a ΔRNA > 0; and 109 a ΔRNA < 0, showing increased stability of RNA secondary structure. For both values, whenever a single variant was located on multiple transcripts, the extreme value was considered.

*5utr* main limitations are that it works only on single nucleotide substitutions (SNVs), and that some annotations are available only for the nearest 100 bp to CDS.

***MORFEE*** (Mutated upstream ORF dEtEction) [31] is an R package that detects and annotates SNVs leading to the formation of upstream translation initiation codons (AUG). This can result in a longer protein (in the case of uAUG in frame with the main CDS with no associated premature stop codon) or in an entirely upstream or overlapping uORF (in the case of a 5′UTR variant associated with a stop codon prior to the main CDS or within it, respectively). After annotation with ANNOVAR [50], 5′UTR variants are filtered, and the GENCODE database is exploited to extract transcripts information and generate the complementary DNA sequences. On this basis, *MORFEE* can determine whether an SNV causes the formation of a new upstream ATG sequence and annotates (i) the distance between the reference ATG and the newly created ATG (uORF-CDS distance), (ii) the reading frame shift (0, 1, 2), and (iii) the predicted uORF length (the distance separating the new ATG from the first stop codon in frame). Overall, *MORFEE* recognized 22 pathogenic events (14 uATG events and 8 uSTOP events) in the 591 variants annotated by Annovar as 5′UTR variants.

Comparing the three tools, MORFEE is limited to annotating variants that create uAUGs only, whereas 5utr and UTRannotator also report the disruption of the existing stop codon of uORFs, the creation of new ones and whether the variant makes the Kozak consensus sequence weaker or stronger. In addition, 5utr annotates the change in predicted translational efficiency [64] and minimum free energy of the secondary structure [65].

**Figure 2:**
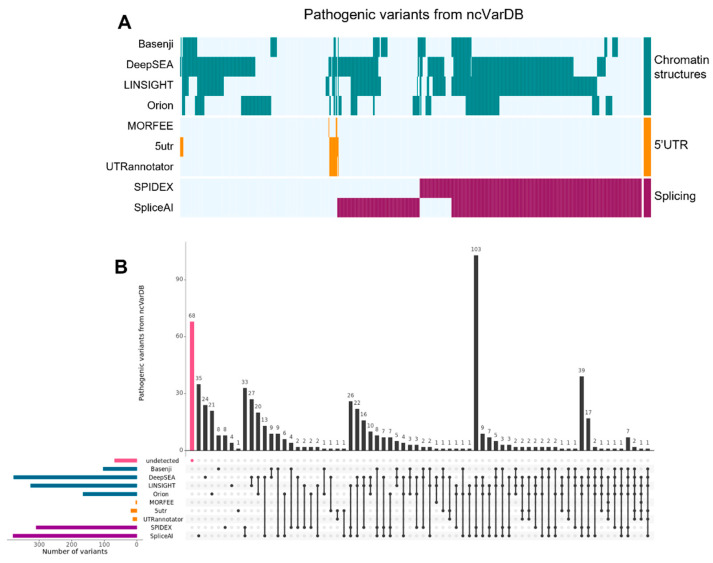
Pathogenic ncVarDB variants (excluding those on chrM) correctly marked as pathogenic by the listed tools. (**A**) The heatmap shows the pathogenic variants (y-axis) detected by each tool (x-axis). A cell is colored whenever a variant is correctly identified as pathogenic. The color scale represents the genomic feature considered by the tools: green, NCVs affecting chromatin structures; orange, variants in 5′UTRs; purple, variants affecting the splicing. (**B**) Upset plot showing the co-occurrence of pathogenic non-coding variants from ncVarDB identified by each tool. The horizontal bars show the total number of pathogenic non-coding variants detected by each tool. The vertical bars show the occurrence or co-occurrence of pathogenic non-coding variants identified through every combination of tools, and the number at the top identifies the cardinality of the set. The black dots and lines show the combination of tools that make up each set.

The computational performance of the three tools is reported in Table 3. Knowledge-based performance (confusion matrix and related indexes) were similar for the three methods, which show quite high specificity and accuracy, whereas insufficient sensitivity and precision. The distribution and overlap of results related to the correct identification of pathogenic variants (referred to ncVarDB data, excluding chrM) are reported in Figure 2. On this basis, *5utr* and *UTRannotator* show overlapping results and a higher number of correctly classified NCV and higher sensitivity than *Morfee*. In total, 19 pathogenic variants in 5′UTR were correctly detected, 17 being detected by multiple tools, while only 2 were specifically discovered by 5′UTR dedicated tools (Figure 2B).

### 3.3. Tools for Identifying NCV Affecting Splicing Sites

The precursor mRNA obtained during transcription has to be spliced into mRNA. During the splicing process, exons are joined together after removing introns. This complex procedure is performed by the spliceosome in eukaryotes and driven by specific sequences and auxiliary splicing of cis elements, namely ss-recognition promoting ‘intronic/exonic splicing enhancers’ (ISEs/ESEs) and ss-recognition repressing ‘intronic/exonic splicing silencers’ (ISSs/ESSs). Canonical splice sites (CSSs) are usually marked, at either exons’ sides, by the canonical sequences ‘AG’ (upstream acceptor sites, 3′ splice site) and ‘GT’ (downstream donor sites, 5′ splice site). These motifs are embedded within consensus splice site recognition sequences (mainly ‘CAG’ and ‘AGGTAAGT’, respectively) still under purifying selection [60]. Regions surrounding canonical splice-sites show constraints similar to protein-truncating and missense variants [66]. Variants affecting the essential sequences GT (at position D+1 and D+2) or AG (at position A-1 and A-2) will lead to the disruption of the consensus sequence, thus resulting in the impaired recognition of the splice site by the splicing machinery. Many mutations not directly affecting the canonical sequences would lead to the same result. Splicing defects within genes are estimated to cause at least 10% of rare pathogenic genetic variants [67] and have been identified as a major source of Mendelian diseases [68,69]. They can be found in CSSs and splice motifs within deep intronic regions (>50 nt from exon-intron boundaries). For example, substitutions within introns may create novel acceptor or donor splice sites recognized by the spliceosome, leading to the inclusion of a portion of the intron in the mature transcript. Less common variants result in the establishment of either new exons within the intron or novel regulatory elements, such as splicing enhancers [70]. Other variants may cause exon skipping, extension, or truncation, leading to the alteration of reading frames or even driving nonsense-mediated decay.

Variants within modulating sequences of alternatively spliced genes may abolish gene function or alter the balance of distinct isoforms, wiping out those depending on the disrupted site [71].

Two of the most commonly used tools for annotating NCV in splicing regions were reviewed here, one leaning on VEP (*SpliceAI*) and one on ANNOVAR (*SPIDEX*).

***SpliceAI* [35]** is an open-source, artificial intelligence-based software freely released by Illumina, which enables the prediction of non-coding variants that disrupt the conventional patterning of exons and introns. It relies on a 32-layer deep neural network exploiting a 10,000-nucleotide window, thus allowing it to use both short-range characteristics (i.e., GT and AG dinucleotides, etc.) as well as long-range features (i.e., exon–intron length, nucleosome positioning, etc.) for exon definition [35,70,72]. The model analyzes the impact of input variants on the splicing, considering all bases within 50 bp from the variant on the pre-mRNA transcript, and returns the surrounding residue with the most significant predicted splicing alteration caused by the variant. In particular, *SpliceAI* outputs the score difference (Delta score, DS) between the predicted exon-intron boundaries in the reference and the alternative pre-mRNA transcript sequence. The output prediction score reflects the splicing-altering probability of a genetic variant (from 0 to 1), which is higher for variants affecting the splicing patterns of a large fraction of gene transcripts and is correlated to the validation rates in the RNA-seq data. For each input variant, *SpliceAI* retrieves the associated gene symbol, DS and delta positions (DP), acceptor gain (AG), acceptor loss (AL), donor gain (DG), and donor loss (DL), specifying the location where splicing differs from the reference (positive values result for the downstream region of the variant, and negative ones for the upstream region). *SpliceAI* authors suggest using 0.5 as the cutoff to distinguish true predicted splice-altering variants; 0.2 would lead to a high recall, while 0.8 is recommended to achieve higher precision values. *SpliceAI* can be run in a standalone mode or with the available pre-scored list for the VEP plugin, which could represent an advantage for VEP users. The pre-computed set contains scores greater than 0.1 for all possible SNVs and 1–4 bases indels within genes defined by GENCODE. Due to the time-consumption of the standalone *SpliceAI* software (analysis of Chr20 took 38 h using a single core and 14.5 h using parallel execution on multiple cores), performance analyses were run exploiting the pre-scored dataset. Out of the 86,132 variants provided, *SpliceAI* scored 34,122 variants affecting the splicing process. Most of these were scored for only 1 gene (27,694 variants, which represent 81.16% of the total scored variants). Moreover, 5279 variants were scored for 2 genes, 867 for 3 genes, 202 for 4 genes, 44 for 5 genes, 16 for 6 genes, 15 for 7 genes, 2 for 8 genes, and 3 for 9 genes. Focusing on the scores provided in default mode, the computation of DS for (i) acceptor gain resulted in 269 variants with 0.5 < DS < 0.8 and 534 variants showing 0.2 < DS < 0.5, (ii) acceptor loss resulted in 349 with 0.5 < DS < 0.8 and 420 with 0.2 < DS < 0.5, and (iii) donor gain resulted in 541 with 0.5 < DS < 0.8 and 739 with 0.2 < DS < 0.5; (iv) donor loss in 667 with 0.5 < DS < 0.8 and 730 with 0.2 < DS < 0.5.

Major limits of *SpliceAI* are the exclusion of variants within 5 kb from the chromosome ends and the focus on SNPs within 50 bp from a canonical exon.

***SPIDEX* [34]** is a database of pre-computed scores generated by a machine-learning approach that evaluates how strongly genetic variants potentially affect an RNA splicing event. This is measured using the Percentage of Spliced-In (PSI) metric, which evaluates whether a splicing isoform is enriched in the presence of a variant. Scores cover all synonymous, missense, and nonsense exonic SNVs, as well as intronic SNVs that are in proximity of splice junctions. *SPIDEX* annotation is available through the ANNOVAR framework [50]. The software returns the gene supposedly affected by the variant, together with two scores: dpsi_max_tissue, a percentage indicating the maximum value, across tissues, of delta PSI (i.e., the predicted change of PSI due to the variant), and dpsi_zscore, the z-score of the former, relative to the distribution of delta PSI due to common SNPs.

The choice of thresholds useful to determine when a variant is predicted to alter splicing is problem-dependent. Nevertheless, Butkiewicz et al. [73] hypothesized that a |z-score| > 2 could indicate a high likelihood of splicing disruption. Over the 86,132 variants in the reference VCF, 3317 were annotated with a SPIDEX score, and 537 had a |z-score| > 2.

A possible limitation of the tool is its ability to analyze only intronic SNVs located up to 300 nt from the splice junctions.

Apart from using different algorithms, the main difference between the two programs lies in the width of the region flanking the variant used for training the model. SpliceAI uses a large window of 10,000 nucleotides to identify long-range features that may affect the spliceosome, whereas SPIDEX is trained on sequence features extracted from each exon and its neighboring introns and exons.

The performance of tools for interpreting variants in splicing regions is reported in Table 3. They are similar both in specificity and precision. *Spidex* appears faster than *SpliceAI*, although the latter can detect a major number of variants. A total of 44 variants located in splicing regions were specifically identified by dedicated tools (35 by *SpliceAI*, 8 by *Spidex*, and 1 by both), while the other 348 were effectively detected also by the other tools (Figure 2).

### 3.4. Tools for Identifying NCV Affecting Genome Accessibility and Mutation Intolerance

Accessible chromatin loci across the genome are called ‘open chromatin regions’ (OCRs). These nucleosome-depleted regions can be bound by protein factors and change dynamically [74] in response to external stimuli and developmental cues, playing crucial roles, among the others, in gene transcription [73]. OCRs containing cis-elements able to be bound by transcription factors are well conserved among eukaryotes. Genetic lesions in these loci can contribute to complex human diseases by re-modulating gene expression and disrupting finely tuned transcriptional networks [3]. Two interesting aspects of the higher-order structures are the so-called ‘topologically associating domains’ (TADs) and the high number of intra-domain interactions. Mutations at TAD boundaries could lead to the rewiring of cis interactions causing genes ectopic expression [75,76,77]. It has been shown that, in patients with presumed genetic diseases, regions intolerant to mutations are enriched for non-coding de-novo pathogenic variants [32]. It has also been highlighted that conservation, indicating that a sequence has been maintained by natural selection, and intolerance seem tightly connected concepts, since ultra-conserved non-coding elements appeared as the most intolerant regions in the human genome [29].

A set of software programs have been developed to deal with higher-order structures, that are becoming crucial for interpreting WGS data.

An example is ***DeepSEA [29]***, a deep-learning-based algorithm that aims at predicting the functional impact of non-coding variants on transcription factor binding sites, DNase I hypersensitivity domains (DHSs), and histone marks, using sequence information alone and with single-nucleotide sensitivity. This represents a challenging task, requiring the accurate modeling of the complex interactions between sequence context, TF binding, and histone modifications. To compute reliable sequence-based predictions, *DeepSEA* uses three main levels of information: (i) a 1 kb genomic region surrounding the variant position; (ii) flexible modeling of the complex interactions between sequences, TFs, DHSs, and histone marks; (iii) joint learning of chromatin factors sharing the same predictive features to increase the computational efficiency. For each input variant, *DeepSEA* computes a functional significance score based on chromatin effect predictions and evolutionary information-derived scores. A functional significance score < 0.01 was considered to define pathogenic variants, as it was the most stringent threshold in the summary output file. Multiallelic variants must be removed before running *DeepSEA*.

Another selected tool is ***Orion [32]***, which detects non-coding regions depleted of variations, suggesting them as intolerant to mutations and subject to purifying selection in the human lineage. *Orion* scans the whole genome and, for each position, it calculates a score as the difference between the observed and expected site-frequency spectrum (SFS) in a window of 501 bp, where SFS is a statistic measure used in population genetics to describe the distribution of allele frequencies across loci in a population. A higher score indicates a more intolerant region and, consequently, a potentially more pathogenic variant. In addition to the software to calculate the score [21], *Orion* provides a pre-computed set of scores, available to directly annotate the variants file through *Vcfanno* annotation software [78]. Among the 86,132 input variants, 41,269 were annotated with an *Orion* score, ranging from a maximum of 0.11607 (rs2979626) to a minimum of −8.87495 (rs561980359). Since a threshold for assuming pathogenicity is not provided, an empirical approach was applied, considering that exons (which tend to be intolerant) typically have a mean score value of −0.174, introns of −0.325, and protein-coding regions of −0.262. Therefore, damaging variants were considered when the score > −0.174. *Orion*’s major limit consists in the absence of pre-computed scores, which can greatly reduce computational time for repetitive regions and sex chromosomes, disabling any chance of finding possible pathogenic variants in those genomic locations.

***LINSIGHT [30]***, the updated version of fitCons [79], provides the ‘fitness consequence’ scores, used as evolution-based measures of potential genomic function. In particular, fitCons aggregates information on natural selection obtained from the INSIGHT (Inference of Natural Selection from Interspersed Genomically coHerent elemenTs) evolutionary model [75,76]. For sites under negative selection, thus less likely to experience a variation in the human lineage, the presence of an SNV will be associated with a low frequency of segregation due to selection. Based on the same concept, *LINSIGHT* (Linear INSIGHT) improved speed, scalability, genomic resolution, and prediction power by coupling the probabilistic INSIGHT model to a generalized linear model for genomic features, avoiding the need for discretization. The tool provides pre-computed *LINSIGHT* scores, which can be employed to estimate negative selection regions in the human genome. The tool could annotate 7252 variants out of the 86,132 in the input VCF. According to the related publication, a score >0.8 should be associated with a strong selection; therefore, variants localized in regions with such scores have been considered pathogenic. Of note, the tool is based on the assumption that natural selection occurred in the past and provides useful information about phenotypic importance in the present time. This approach leads to some limitations, as, in this light, variants influencing clinical traits that do not show signs of historical purifying selection (e.g., those influencing phenotypes dependent on the modern human environment) could be difficult to study. Moreover, traits related to highly epistatic loci or the contribution of numerous loci could be difficult to attribute to single nucleotides.

***Basenji [19]***, the successor of Basset, is a deep convolutional neural network (CNN)-based approach. *Basenji* allows: (i) training deep CNN to predict regulatory activity along very long chromosome-scale DNA sequences; (ii) score variants according to their predicted influence on regulatory activity; (iii) annotating the distal regulatory elements that influence gene activity and the specific nucleotides that drive regulatory element function. For each variant, two scores are retrieved: SAD (SNP Activity Difference) and SED (SNP expression difference, which requires additional file input). SAD represents the difference between the predicted accessibility of the alternative and the reference alleles. Thus, higher positive SAD indicates higher predicted chromatin accessibility on the alternative allele than on the reference one [80], and the higher the absolute value, the higher the impact of the considered variant on accessibility. For *Basenji*, the pre-trained model offered by developers (downloadable from GitHub) was used. *Basenji* annotated all 86,132 variants contained in the VCF input file. It is relevant to note that, for each variant, 5313 SAD scores were computed, referring to different types of cell and experimental techniques (CAGE, DNase, and chip) [81]. The gold standard for analyzing results is to focus on SAD scores from training datasets relevant to the cell type or tissue of interest. However, since tests carried out in this review are general purpose, human embryonic stem cells (hESC) were considered. Since the resulting values were highly variable, a predefined threshold to identify relevant variants was difficult to detect. Thus, z-scores were computed for all variants, and |z-score| > 1.96 was considered relevant, allowing the identification of the elements belonging to the extreme 5% of the area under the normal curve. Overall, 1610 variants exceeding the fixed threshold were detected. A recognized approach for threshold definition represents the main disadvantage of *Basenji* and could easily lead to unreliable results.

The methodologies applied by the tools in this category are quite diverse. Basenji and DeepSEA are sequence-based methods. Basenji is trained on chromatin accessibility profiles to predict the impact of genomic variants on gene expression, while DeepSEA is based on a wide range of chromatin profiles to estimate the effects of sequence alterations on chromatin accessibility. LINSIGHT belongs instead to the class of evolutionary-based methods. It combines functional and comparative genomic data to predict the effects of non-coding variants on fitness. Finally, Orion uses population genetics-based methodologies to identify genomic regions that are intolerant to variation and are therefore more likely to be pathogenic when mutated. These approaches, albeit different, are interconnected because chromatin accessibility, evolutionary conservation, and mutation intolerance are highly correlated features [82,83].

The performance of the considered tools for interpreting variants affecting genome accessibility and conserved regions is reported in Table 3. All the considered tools showed good specificity, while only *LINSIGHT* reported acceptable precision, as well as being the fastest. Conversely, *Basenji* could detect the smallest number of TP, resulting to be the slower and less precise tool (Table 3 and Figure 2). Interestingly, *LINSIGHT* and *DeepSEA* show quite a high overlap in variant detection (277 variants commonly detected, of which 27 were specifically identified by these two tools) although their aims in principle are quite different, *LINSIGHT* being focused on the evolutionary context, and *DeepSEA* on chromatin structure aspects. A minor overlap is identifiable between the results of *LINSIGHT* and *Orion*, although dedicated to similar aspects (respectively, regions that are conserved and intolerant to variations).

### 3.5. Phenotype-Based Functional Prediction Tool

Among the general purpose “investigator” tools, ***Genomiser*** [26] is the only one able to interpret and associate non-coding variants to specific Mendelian disorders. It is an Exomiser-based [84] whole–genome analysis framework able to score the relevance of variations in the non-coding genome and associate them with specific Mendelian diseases while exploiting the Human Phenotype Ontology [85] as input data. It relies on a customizable configuration file containing all the steps to be performed, the input VCF file, the pedigree (for multiple samples), the patient’s HPO terms (advised), and the inheritance model (if known). Each variant is scored according to CADD or ReMM score [86,87] (from 0 for non-deleterious to 1 for deleterious), allele frequency, regulatory sequences, chromosomal topological domains, and its phenotypic relevance in relation to the disease. Scores can be prioritized through phenotype similarity algorithms, assessing variants’ likelihood to contribute to the pathological phenotype. *Genomiser* can be run with or without HPO terms. In this latter case, results are based on ReMM-predicted pathogenicity and the allele frequency of the variant in reference databases, their rank relying on their likelihood of pathogenicity.

The output includes the associated genes and, for each variant, (i) the available pathogenicity scores (i.e., CADD, Polyphen, Mutationtaster, Sift, ReMM), (ii) the Exomiser variant score, which predicts a measure of rarity and pathogenicity of the variant (0 for probably benign, 1 for probably pathogenic), (iii) the Exomiser gene-phenotype score, based on the phenotypic similarity between the given HPO terms and rare diseases known to be associated with the gene in OMIM or Orphanet (from 0, if no phenotype information is provided, to 1), (iv) the Exomiser gene-variant score, which states how likely the considered variant acts on the predicted gene, and (v) the Exomiser gene combined score, used for the prioritization process, ranging from 0 to 1 [84,88]. Initial tests were performed on Chr20 variants, without relying on HPO terms since data belonged to a healthy individual, to evaluate the tool’s performance in the simplest conditions. In this case, 380 out of 78,186 variants contained in the considered VCF file were marked as relevant, 364 of which lay in non-coding regions. To exploit *Genomiser*’s ability to detect the variants’ impact on the provided HPO phenotype, it was also run after enriching the Chr20 VCF file with 6 variants (two in splice regions, two upstream a gene, one in 5′UTR, and one in an intron) selected from ncVarDB pathogenic set, causing independent pathologies. Associated HPO terms were also provided to detect the ability of the tool to correctly identify the additional variants as the cause of the disease. *Genomiser* was run 6 times (1 for each variant and associated pathology), and, in all cases, it was able to detect the variant of interest as contributing to the HPO term-related pathology, ranking it on top of the list (1st or 2nd position). Overall, *Genomiser* took less than 10 min to provide the output.

## 4. Discussion

Bioinformatics tools currently available to predict the effect of genetic variants in the human genome are heavily biased towards the protein-coding regions due to the wider knowledge related to genes and protein function. Despite the scarce attention gained in the past, non-coding variants may affect gene function, thus playing a role in human diseases. Interpreting the effects of NCVs is a new and difficult challenge, which needs to be afforded with the support of advanced bioinformatics software programs. In this review, tools for non-coding variants’ effect interpretation were evaluated, and their performance was tested on a computational and information basis. In particular, tools for interpreting variants falling in UTRs, splicing, and intolerant regions, other than those affecting the genome accessibility, were taken into account, tested, and compared. 

Other non-coding regions of the human genome could be modified by variants potentially affecting the system’s physiology. Among them, non-coding RNAs (ncRNAs) and proximal/distal cis-regulatory elements are of particular interest. From a process regulation perspective, housekeeping and regulatory ncRNAs exist. The first are abundant and ubiquitous in cells and primarily regulate generic cellular functions, whereas the second ones act as regulators of gene expression at epigenetic, transcriptional, and post-transcriptional levels [74,89,90,91]. Gene expression regulation is also strictly monitored by proximal and distal cis-regulatory elements, characterized by defined histone marks to enable their identification and to regulate transcription activation based on timing, tissue, and associated genes. Proximal elements mainly include gene promoters, whereas distal elements refer to enhancers, typically found in DNaseI hypersensitive sites surrounded by nucleosomes marked by H3K4me1 and H3K27ac, and transcription factors [92,93]. Promoters and enhancers include specific motifs in their sequence, which are recognized and bound by distinct transcription factors, and, probably, other non-coding elements. This should be considered when investigating the pathogenicity of variants on such elements [94,95,96]. Other elements in non-coding regions are CpG islands, regions of at least 200 bp, with a GC amount greater than 50%, and an observed-to-expected CpG ratio greater than 60% [97]. Mutations at CpG islands could affect promoter activity, causing, for example, stable silencing or, conversely, the constitutive expression of genes [98].

It should be noted that, at the moment, variants at these sites cannot be functionally annotated by specific tools but only by general-purpose ones that are potentially able to deal with variants over the entire genome.

To carry out quantitative analysis, a controlled scenario was identified, exploiting variants identified in Chr20 of a healthy individual, enriched by the set of known benign and pathogenic variants available through ncVarDB. The full dataset was helpful when determining computational performance, while the ncVarDB subset was crucial for calculating specificity, precision, sensitivity, and accuracy (Table 3). Specificity, which defines how good a test is in identifying true negatives, was >0.8 for all tools, whereas the other performance features spanned from extremely low to extremely high values. Overall, the entire group of examined tools contributed to the functional annotation of most of the 721 pathogenic variants provided by ncVarDB (Figure 2). Among them, 596 (82.66%) were correctly annotated as pathogenic by at least one tool. Regarding the 125 pathogenic variants not annotated correctly, 57 belonged to the mitochondrial chromosome (not considered by the majority of tools), whereas 11, although annotated as pathogenic by ncvarDB, are reported in ClinVar [99] with conflicting or uncertain interpretations. The remaining 57 (7.9%) pathogenic variants were not correctly classified by any tool considered in this review. Relying on the fact that each evaluated software contributed to the variant identification with a set of uniquely detected variants, the overall approach we could suggest when dealing with the analysis of non-coding regions consists of the integration of outputs from multiple methods, as also reported by Liu et al. [100]. Figure 2 is highly representative, from both qualitative (Figure 2A) and quantitative (Figure 2B) points of this concept view, underlying the need to combine more than one tool to both functionally annotate the vast majority of variants and to strengthen the reliability of prediction. However, most variants are correctly detected by wide-spectrum programs such as *DeepSea*, *LINSIGHT*, and *SpliceAI*, and their combination may constitute a good compromise.

Another consideration concerns the computational efforts: the present study was carried out on a single chromosome of a single individual. Nevertheless, in WGS, 23 chromosome couples should be analyzed, often in trios. This implies that high-performance computing will be necessary for WGS analysis, and available software should enable the parallel computational paradigm.

Hopefully, in the near future, advances in both molecular biology (some regions of the genome deserve additional research to evaluate the consequences of variants in those sequences) and computational domains (novel bioinformatics algorithms are expected, exploiting cutting-edge technologies) will significantly improve the understanding of non-coding variants, whose impact is still an open challenge in current human genetics.

## Figures and Tables

**Table 1:** Number of variants available in ncVarDB after conversion to hg19.

Category	Pathogenic Variants	Benign Variants
5′UTR	64	639
intergenic	10	100
non-coding RNA	72	738
intronic	539	5389
3′UTR	36	359

**Table 2:** List of the tools considered for non-coding variant analysis.

Scheme	Notes	Aim	Target	Var Type	Ref./ Year	License	Input	Output	Allowed Ref. Genome	Repository/Homepage
**INVESTIGATORS**										
ARVIN	1	Predicting causal non-coding variants. It infers non-coding risk variants starting from known causal variants in gene promoters and enhancers for a number of diseases	SNPs on enhancers	SNVs/ Indels	[18]	free	SNPs in bed format + Enhancer-promoter interaction file + GWAVA features + FunSeq features + Differential expression *p*-values file	Prediction score for a list of snps for being risk or non-risk snps	Hg19	https://github.com/gaolong/arvin
Basenji	Tested	Annotating every mutation in the genome with its influence on present chromatin accessibility and latent potential for accessibility	Epigenetic regions	SNVs	[19]	free	VCF	SNP activity difference (SAD) score—difference between the predicted accessibility of the alternative and the reference alleles	Hg19	https://github.com/calico/basenji
DeepBind	1	Predicting the sequence specificities of DNA- and RNA-binding proteins by deep learning	Binding sites	SNVs	[20]	free	(1) a list of model IDs, and (2) a list of DNA/RNA sequences	Weighted ensemble of position weight matrices or a ‘mutation map’ that indicates how variations affect binding within a specific sequence	Hg19	https://github.com/jisraeli/DeepBind.git
DeepSEA	Tested	Predicting genomic variant effects on a wide range of chromatin features at the variant position: transcription factors binding, DNase I hypersensitive sites, and histone marks in multiple human cell types	General purpose	SNVs/Indels	[21]	free	VCF/FASTA/BED (single file)	Multiple files analyzing chromatin feature probabilities and a functional significance score	Hg19	http://deepsea.princeton.edu/help/
DeltaSVM	1	Quantifying the effect of variants in regulatory non-coding regions	Regulatory non-coding regions	SNVs	[22]	free	Reference FASTA file (19 bp sequences centered at the SNPs with reference alleles) + Alternate FASTA file (19 bp sequences centered at the SNPs with alternate alleles) + SVM weight file (available for download from the webpage)	DeltaSVM scores, allowing the prediction of risk-conferring SNPs	Hg19, Hg38	http://www.beerlab.org/deltasvm/
Epossum2	1	Predicting the impact of DNA variants on transcription factor binding	Binding sites	SNVs/Indels	-	free	Single/few variants (in VCF-like format), but does not accept a VCF file directly + one or more TFs	For each variant, a blue/red box indicates the likelihood of increased/reduced TF binding	Hg19	https://www.genecascade.org/ePOSSUM2/
FUN-LDA	1	Predicting Tissue-Specific Functional Effects of Non-coding Variation	General purpose	SNVs/Indels	[23]	free	.csv, .txt or .gz file without header (chr, hg19 coordinate or rs number), <100,000 var	FUN-LDA scores for each Roadmap tissue + Eigen and Eigen-PC raw and phred scores)	Hg19	https://github.com/cran/FUNLDA
Geneyx (former Tgex’)	2	Working with GeneHancer and VarElect, it translates the finding of a variant in a non-coding region into a variant-to-gene-to-phenotype annotation	SNVs in enhancers, promoters, and ncRNA genes	SV/SNVs	[24]	license	VCF	Report containing prioritized variants, together with their annotation and analysis	Hg19	https://geneyx.com/geneyxanalysis/
GenoCanyon	1	Inferring the functional potential of each position in the human genome	Conserved regions	SNV	[25]	free	Chromosomal region/prediction score for the region; pre-computed scores are available	GenoCanyon score stating whether a genomic locus is functional or non-functional	Hg19	http://zhaocenter.org/GenoCanyon_FAQ.html
Genomiser	Tested	Scoring the relevance of variation in the non-coding genome, and also associating regulatory variants to specific Mendelian diseases	General purpose	SNVs/Indels	[26]	free	VCF, PED-file (only for multiple samples in one VCF), your patient’s HPO terms (use the HPO-Browser to find terms), the inheritance model if known, the output prefix for your output files	Annotates, filters, and prioritizes likely causative variants, formulating a score	Hg19	https://github.com/exomiser/Exomiser
Human Splicing Finder	2	Identifying all splicing signals, including acceptor and donor splice sites, branch points, and auxiliary splicing signals (ESE and ESS)	Splicing	SNVs/Indels	[27]	license	Single mutations/VCF, submitted through the website by directly submitting a VCF file through an API	Pathogenicity prediction for any mutation potentially affecting splicing	Hg19	https://www.genomnis.com/the-system-1
INFERNO	1	Inferring the molecular mechanisms of causal non-coding variants	General purpose	SNVs/Indels	[28]	free	GWAS/TSV (chromosome\t rsID \t region name\t position) maximum of 8 Mb/rsIDs	Several files containing relevant tissue contexts, target genes, and downstream biological processes affected by functional variants	Hg19	https://bitbucket.org/wanglab-upenn/inferno/src/master/
JARVIS	3	Prioritizing non-coding variants in whole genomes, using human-lineage purifying selection features and primary sequence context	Conserved regions	SNVs	[29]	free	VCF and pre-calculated JARVIS scores gwRVIS—genome-wide intolerance to variation score	gwRVIS—genome-wide intolerance to variation score	Hg19, Hg38	https://github.com/astrazeneca-cgr-publications/JARVIS
LINSIGHT	Tested	Improving the prediction of non-coding nucleotide sites at which mutations are likely to have deleterious fitness consequences	Conserved regions	SNVs	[30]	free	VCF and Precomputed LINSIGHT scores	LINSIGHT score, which measures the probability of negative selection on non-coding sites	Hg19	https://github.com/CshlSiepelLab/LINSIGHT
MORFEE	Tested	Detecting variants creating new uORF (new uAUG)	5′UTR	SNVs	[31]	free	VCF (ANNOVAR-annotated)	MORFEE annotation reporting the effect of the variant on 5′UTR	Hg19	https://github.com/daissi/MORFEE
Orion	Tested	Detecting regions of the non-coding genome that are depleted of variation, suggesting that the regions are intolerant to mutations and subject to purifying selection in the human lineage	Conserved regions	SNVs	[32]	free	One or more gVCF files, a summary by position for all samples of either read depth or genotype quality (GQ), and a file containing mutation rates; pre-computed scores exist	Orion score, with higher values corresponding to a higher degree of intolerance	Hg19	https://github.com/igm-team/orion-public
RegulomeDB	1	Annotating SNPs with known and predicted regulatory elements in the intergenic regions	Regions of DNase hypersensitivity, binding sites of TF, promoter regions	SNVs	[33]	free	dbSNP IDs/0-based coordinates in batch	Graphic output + table containing experiments (often localized according to tissues) regarding various aspects (chromatin state, accessibility, motifs, chip data, qtl)	Hg19	https://regulomedb.org/regulome-search/
SPIDEX	Tested	Assessing whether a variant causes dysregulation of a splicing event	Splicing	SNVs	[34]	free	SPANR: maximum of 40 SNV at a time; SPIDEX: VCF of SNV	Score evaluating whether a certain splicing isoform is more enriched under the presence/absence of a given variant	Hg19	http://download.openbioinformatics.org/spidex_download_form.php
SpliceAI	Tested	Identifying variants impacting splice sites	Splicing	SNVs/Indels	[35]	free	VCF	Delta score, highlighting the probability of the variant being splice-altering	Hg19, Hg38	https://github.com/Illumina/SpliceAI
5utr	Tested	Providing different annotations relevant to 5′UTR (untranslated region) variants	5′UTR	SNVs	-	free	VCF	Annotation reporting the effect of the variant on 5′UTR	Hg19, Hg38	https://github.com/leklab/5utr
UTRannotator	Tested	Annotating high-impact five prime UTR variants either creating new upstream ORFs or disrupting existing upstream ORFs	5′UTR	SNVs/Indels	[36]	free	VCF	Annotation reporting the effect of the variant on 5′UTR	Hg19, Hg38	https://github.com/ImperialCardioGenetics/UTRannotator
VarElect GeneHancer	1, 2	Inferring direct and indirect links between genes (or enhancers and promoters included in GeneHancer) and phenotypes—GeneHancer is a database of human regulatory elements (enhancers and promoters) and their inferred target genes	Enhancers	N.A.	[37,38]	license	Gene symbols and phenotypes	List of input genes with a score that tells how much each gene is associated with the requested phenotype	Hg19, Hg38	https://github.com/ucscGenomeBrowser/kent/blob/master/src/hg/makeDb/doc/geneHancer.txt
**INTEGRATORS**										
CADD		Scoring the deleteriousness of single nucleotide variants as well as insertion/deletions variants	General purpose	SNVs/Indels	[39]	Free *	VCF or .tsv.gz	CADD score, measuring the deleteriousness of SNVs and indels	Hg19, Hg38	https://github.com/kircherlab/CADD-scripts
DANN		Predict pathogenicity of SNVs and indels using deep neural network	General purpose	SNVs/Indels	[40]	free pyTorch implementation	VCF or .tsv.gz	DANN score, measuring the pathogenicity of SNVs	N.A.	https://cbcl.ics.uci.edu/public_data/DANN/
DVAR		Genome-wide functional scores	General purpose	SNVs/Indels	[41]	free	.tsv (chromosome, position, the ref nucleotides, the obs nucleotides, and the rs number)	Produced functional cluster labels and scores the importance of each variant	Hg19	https://www.vumc.org/cgg/dvar
Eigen v1.0		Eigen uses a variety of functional annotations in both coding and non-coding regions and combines them into one single measure of functional importance	General purpose	SNVs	[42]	free	VCF	Eigen score, measuring how functional the variant is	Hg19, Hg38	http://www.columbia.edu/~ii2135/eigen.html
FATHMM-XF (old -MKL)		Functional predictor for SNVs	SNVs	SNVs	[43]	free	VCF or csv (chr, pos, ref n, mut n)	Score highlighting the variant pathogenicity	Hg19, Hg38	https://github.com/HAShihab/fathmm-MKL
GWAVA		Predicting the functional impact of non-coding genetic variants based on a wide range of annotations of non-coding elements, along with genome-wide properties such as evolutionary conservation and GC-content	General purpose	SNVs/Indels	[44]	free	Multiple variant identifiers (in BED format) or chromosomal regions	GWAVA score, with higher scores indicating variants predicted as more likely to be functional	Hg19	https://www.sanger.ac.uk/tool/gwava/
IW-Scoring		Scoring (integrates in a weighted way the outputs of other software, chosen by the user from a list)	Non-coding variations	SNVs/Indels	[45]	free	In batches up to 100 K SNPs/InDels (also VCF format, but not exclusively)	Two separate linear weighted functional scoring schemas for known and novel variations, respectively, which differentiate functionally significant variations from others	Hg19	https://snp-nexus.org/IW-Scoring/index.html
PAFA		Genome-wide functional scores	General purpose	SNVs/Indels	[46]	free	VCF (<100.000 var) max 2 Mb	Prioritization of variants + functional score	Hg19, Hg38	http://159.226.67.237:8080/pafa/
PINES		Predicting the functional impact of non-coding variants by integrating epigenetic annotations in a phenotype-dependent manner	General purpose	SNVs	[47]	free	List of intronic or intergenic variants one rs per line	Identification and prioritization of functional non-coding SNPs	Hg19	https://github.com/PINES-scoring/PINES
RegulationSpotter		Integrating data from various sources to show whether a variant lies within a regulatory region and has the potential to impair gene expression	Intolerant regions	SNVs/Indels	[48]	free	Single variant or VCF format	Summary table with a graphical matrix depicting key aspects of all analyzed variants	Hg19	https://www.regulationspotter.org/
**AGGREGATORS**										
Alamut (Batch)		Annotator	General purpose	SNVs/Indels	[49]	license	VCF, tab-delimited files	Annotated variants	Hg19, Hg38	https://www.interactive-biosoftware.com/alamut-batch/
ANNOVAR		Annotator	General purpose	SNVs/Indels	[50]	free	VCF	Annotated variants	Hg19, Hg38	https://github.com/WGLab/doc-ANNOVAR
BasePlayer		Large-scale discovery tool for genomic variants allowing for complex comparative variant analyses	General purpose	SNVs/Indels	[51]	free	BAM or VCF (+BED)	Graphical user interface for variants visualization (built-in genome browser, interactive variant analysis, and data integration tracks)	Hg38	https://github.com/rkataine/BasePlayer
RegBase		Integrating non-coding regulatory prediction scores and composite prediction models from existing tools	Non-coding regulatory variants	SNVs	[52]	free *	Chromosome coordinates/query_file.bed	Variants prioritization	Hg19	https://github.com/mulinlab/regBase
SnpEff		Annotating and predicting the effects of genetic variants	General purpose	SNVs/Indels	[53]	free	VCF	Annotated variants	Hg19, Hg38	https://github.com/pcingola/SnpEff
SNPNexus		Variants annotation tool designed to simplify and assist in the selection and prioritization of known and novel genomic alterations	General purpose	SNVs/Indels	[54]	free *	Single and batch	Annotated variants	Hg19, Hg38	https://www.snp-nexus.org/v4/
VarAFT		Provides experiments’ quality, annotates, and allows the filtration of VCF files; annotates and pinpoints human disease-causing mutations through access to multiple layers of information	General purpose	SNVs/Indels	[55]	free	VCF or ANN 4.1	Graphical user interface that allows the simultaneous annotation, filtration, and breadth and depth of coverage analysis	Hg19, Hg38	https://varaft.eu/
VEP Predictor		Annotator (but specific modules for non-coding variants exist)	General purpose	SNVs/Indels	[17]	free	VCF, rsID or HGVS notations	Annotated variants	Hg19, Hg38	https://github.com/Ensembl/ensembl-vep

Note: The ‘Notes’ column reports, for each ‘investigator’ tool, the reason for its exclusion from the test: 1, the tool does not accept VCF as input; 2, the tool is not freely available; 3: tool not available. (*) free for academic or non-commercial use.

**Table 3:** Performance for each selected tool, grouped by category.

Tool	Number of Annotated Variants from ncVarDB	Computational Performance	Parallel Paradigm	TP/FP/TN/FN	Specificity	Sensitivity	Precision	Accuracy
**UTRs**								
UTRannotator	478	1 m–10 m	yes	13/24/396/45	0.943	0.224	0.351	0.856
5utr	478	1 m–10 m	yes	20/78/342/38	0.814	0.345	0.204	0.757
MORFEE	433	10 m–30 m	no	3/14/368/48	0.963	0.059	0.176	0.857
**Splicing sites**								
SpliceAI	4854	30 m–1 h	yes	389/1/4249/n.a.	0.9998	---	0.997	---
SPIDEX	756	<1 m	Yes ^1^	314/19/273/n.a.	0.935	---	0.943	---
**Genome accessibility and mutation intolerance**								
DeepSEA	7879	>1 d	no	388/243/6980/n.a.	0.966	---	0.615	---
Orion	4373	1 m–10 m	Yes ^2^	169/311/3482/n.a.	0.918	---	0.352	---
LINSIGHT	1240	<1 m	Yes ^2^	334/59/676/n.a.	0.9197	---	0.8499	---
Basenji	7946	>1 d (on CPU) 5 h–24 h (on GPU) ^3^	yes	117/1263/5962/n.a.	0.825	---	0.085	---

Note: ^1^ *SPIDEX* annotations are available through ANNOVAR, which supports multi-threading; ^2^ When using pre-computed scores, the annotation relies either on VEP or vcfanno, which both support multi-threading. ^3^ Working on a single CPU, in accordance with the other software tests, the tool required days to complete the run. An additional test on a single GPU took 23 h to generate the final output. n.a.: not available. Computational performance is defined with predefined time intervals: <1 m, 1 m–10 m, 10 m–30 m, 30 m–1 h, 5 h–24 h, >1 d, where m = minute, h = hour, d = day.

## Data Availability

Variants in chromosome 20 were extracted from the VCF file of a sample of Han Chinese ancestry [15] (HG005-NA24631) retrieved from the Genome In A Bottle (GIAB) project [16]. The set of known pathogenic and benign NCVs was obtained from the manually curated ncVarDB [13] database.
